# Editorial: Future of heart valve surgery: enhancing outcomes with innovative technologies

**DOI:** 10.3389/fcvm.2026.1974572

**Published:** 2026-09-04

**Authors:** Omer Dzemali, Theodor Fischlein, Héctor Rodriguez Cetina Biefer

**Affiliations:** 1 Department of Cardiac Surgery, University Hospital Zurich, University of Zurich, Zurich, Switzerland; 2 Department of Cardiac Surgery, City Hospital Zurich, Zurich, Switzerland; 3 Faculty of Medical Sciences, State University of Tetova, Tetova, North Macedonia; 4 Department of Cardiac Surgery, Klinikum Nürnberg, Paracelsus Medical University, Nuremberg, Germany

**Keywords:** bioprosthesis durability, cardiac imaging, heart valve surgery, minimally invasive cardiac surgery, mitral valve repair, risk stratification, transcatheter aortic valve implantation

## Introduction

Valvular heart disease is increasing in prevalence as populations age, while its therapeutic landscape has undergone a remarkable transformation over the past two decades, arguably greater than during the preceding half-century. When many of us trained, treatment of advanced valvular disease was largely synonymous with open-heart surgery, with few meaningful alternatives. Today, the same patient may be offered a minimally invasive repair, a transcatheter implant, or a staged combination of both, and this choice is increasingly guided by imaging and procedural planning tools that did not exist until recently. This Research Topic was assembled to take stock of these developments. The ten included articles cannot capture the entire field, but together they offer a representative view of its current trajectory.

## Aims of the research topic

Of particular interest to us as editors was not any single technical advance, but rather how these advances relate to one another within the broader clinical pathway. A new device or access route is justified only if it produces a measurable benefit to the patient — be it longer survival, fewer reoperations, or a shorter recovery — and only if this benefit is reproducible in routine clinical practice rather than confined to a small number of expert centers. The accepted articles fall into several natural categories. Some address surgical technique, whether the operative access or the repair itself. Others address transcatheter and hybrid treatment strategies. A further group focuses on risk prediction and long-term outcomes, and two contributions return to the imaging and anatomical foundations on which the rest of the field depends. It is also notable what this Research Topic does not contain: no mature clinical application of artificial intelligence is represented here. The digital contributions concern imaging and quantitative measurement rather than autonomous decision-making, which likely reflects current clinical practice more broadly.

## Minimally invasive and reconstructive surgery

Minimally invasive valve surgery is no longer a niche approach; in many high-volume centers, it has become the standard route to the mitral valve. Theodosiadis et al. compared a conventional minimally invasive approach with a fully endoscopic technique, addressing a question relevant to any unit adopting these methods: the extent to which additional procedural complexity is justified by a smaller incision and faster recovery. Valve repair itself continues to improve through incremental refinements that ultimately determine its durability. Nasso et al. report five-year outcomes of a modified technique for sizing artificial chordae in anterior leaflet repair, underscoring a broader principle: the durability of valve-preserving surgery depends precisely on such technical details. Robotic surgery belongs within this same discussion, primarily for its potential to improve reproducibility across cases, although no robotic series is represented in this collection.

## Transcatheter and hybrid therapies

Transcatheter aortic valve implantation has extended into younger and lower-risk patient populations, prompting renewed discussion across nearly every aspect of aortic valve disease management. Three contributions address distinct facets of this shift. Krishnareddigari et al. present propensity-matched single-center data favoring the SAPIEN 3 Ultra RESILIA valve with respect to hemodynamic performance and paravalvular leak, representing one of the incremental improvements that continue to narrow the gap between transcatheter and surgical approaches. Fang et al. examine a more complex clinical scenario — the patient with concomitant aortic stenosis and coronary artery disease — comparing TAVR combined with PCI to surgical aortic valve replacement combined with coronary artery bypass grafting over both short- and longer-term follow-up. Their findings underscore the importance of continued reassessment of heart team decision-making thresholds. Because management does not conclude once the valve is implanted, Zhu et al. investigate antithrombotic therapy following transcatheter mitral repair in patients with atrial fibrillation, an area in which the balance between thrombotic and bleeding risk remains unresolved.

## Risk stratification and long-term outcomes

Novel techniques carry limited clinical value without rigorous evaluation of patient benefit and its durability over time. Hiremath et al. report five-year outcomes for the Dafodil pericardial bioprosthesis. Durability is readily claimed but demonstrated only gradually, and it ultimately informs valve selection in younger patients. Chen et al. investigated left atrial strain as a predictor of outcome in significant aortic valve disease, a parameter that has established its relevance alongside existing risk scores. Hou et al. compare repair with replacement in rheumatic mitral valve disease, a condition largely absent from clinical practice in high-income countries but still associated with substantial morbidity elsewhere, and for which the optimal surgical approach remains unresolved. Such work should not be regarded as secondary to innovation; it constitutes, to a considerable extent, its principal purpose.

## Imaging, anatomy, and the translational foundation

Underlying all of these developments is the foundational work on which they depend. Pouch et al. trace the evolution of aortic valve imaging from three-dimensional energy-integrating CT to four-dimensional photon-counting CT. This advance extends beyond improved spatial resolution: it enables the valve to be characterized as the dynamic structure it is, with implications for both surgical and transcatheter procedures. Strauss et al. raise a consideration that is often underappreciated because it originates from preclinical rather than clinical work: the aortic root anatomy of large-animal models commonly used for device testing differs from the human aortic root in clinically relevant ways. This should be taken into account by investigators designing or validating devices using these models. Should artificial intelligence become integrated into routine valve care in the near future, it will most likely first be applied to the interpretation and quantification of imaging data already acquired in clinical practice, rather than to autonomous clinical decision-making.

## Toward a more personalized and efficient future

Considered together, these contributions convey a consistent message: optimal valve care depends less on any individual technology or operator than on the effective integration of surgical, transcatheter, and imaging strategies for the individual patient. This integration is the responsibility of the heart team, informed by improved risk models and longer-term follow-up data, and it requires a measure of clinical restraint. As newer devices are increasingly implanted in younger patients who may live with them for decades, durability, cost, and equitable access can no longer be regarded as secondary considerations; they must be incorporated into the indication itself.

## Conclusion

We thank the authors and the reviewers whose critical evaluation strengthened these contributions. Together, they provide an accurate account of a field undergoing rapid, if uneven, progress toward valve treatment that is safer, less invasive, and more precisely matched to the individual patient. We hope this collection will be of value to clinicians, researchers, and industry colleagues, and that it will encourage both continued innovation and the rigorous evaluation that must accompany it.

